# Detailed ecological associations of triatomines revealed by metabarcoding and next-generation sequencing: implications for triatomine behavior and *Trypanosoma cruzi* transmission cycles

**DOI:** 10.1038/s41598-018-22455-x

**Published:** 2018-03-07

**Authors:** Eric Dumonteil, Maria-Jesus Ramirez-Sierra, Silvia Pérez-Carrillo, Christian Teh-Poot, Claudia Herrera, Sébastien Gourbière, Etienne Waleckx

**Affiliations:** 1 Department of Tropical Medicine, Vector-Borne and Infectious Disease Research Center, School of Public Health and Tropical Medicine, Tulane University, New Orleans, LA USA; 2 Laboratorio de Parasitología, Centro de Investigaciones Regionales “Dr. Hideyo Noguchi”, Universidad Autónoma de Yucatán, Mérida, Yucatán Mexico; 3 Institut de Modélisation et d’Analyses en Géo-Environnement et Santé, Université de Perpignan Via Domitia, Perpignan, France

## Abstract

*Trypanosoma cruzi* is the agent of Chagas disease, transmitted by hematophagous triatomine vectors. Establishing transmission cycles is key to understand the epidemiology of the disease, but integrative assessments of ecological interactions shaping parasite transmission are still limited. Current approaches also lack sensitivity to assess the full extent of this ecological diversity. Here we developed a metabarcoding approach based on next-generation sequencing to identify triatomine gut microbiome, vertebrate feeding hosts, and parasite diversity and their potential interactions. We detected a dynamic microbiome in *Triatoma dimidiata*, including 23 bacterial orders, which differed according to blood sources. Fourteen vertebrate species served as blood sources, corresponding to domestic, synantropic and sylvatic species, although four (human, dog, cow and mice) accounted for over 50% of blood sources. Importantly, bugs fed on multiple hosts, with up to 11 hosts identified per bug, indicating very frequent host-switching. A high clonal diversity of *T. cruzi* was detected, with up to 20 haplotypes per bug. This analysis provided much greater sensitivity to detect multiple blood meals and multiclonal infections with *T. cruzi*, which should be taken into account to develop transmission networks, and characterize the risk for human infection, eventually leading to a better control of disease transmission.

## Introduction

*Trypanosoma cruzi* is the agent of Chagas disease, a major vector-borne parasitic disease in the Americas. *T. cruzi* parasite transmission from triatomine vectors to vertebrate hosts occurs via the insect’s feces through a complex succession of events, making transmission from vector to vertebrate host rather unlikely^1^. The parasite is nonetheless able to infect a very large variety of mammalian hosts covering many orders, including Marsupialia, Rodentia, Lagomorpha, Chiroptera, Carnivora and Primata. The identification of the host species involved in transmission cycles is key to understand the epidemiology of the disease and the risk for human transmission. The identification of triatomine host feeding patterns and their variations^2–7^ is starting to provide useful information to assess the role of different hosts species as parasite reservoirs or for contributing to increasing the risk for human infection. Separate or overlapping transmission cycles among species communities may also be evidenced^7–10^. Nonetheless, host feeding sources and feeding behavior of triatomines remain poorly understood as current techniques based on direct sequencing of PCR products can only identify the dominant sequence/host in each sample. The addition of a cloning step prior to sequencing allowed detecting up to 3–5 host species in some bugs^4–6^, suggesting that multiple blood meals may be more frequent than previously acknowledged. Such multiple blood meals are key to evaluate the potential for parasite transmission between hosts species.

Similarly, while parasite genetic diversity has been known for a long time and has led to the division of *T. cruzi* into seven discrete typing units (DTUs TcI to TcVI and Tcbat)^11,12^, the geographic distribution of the different DTUs as well as their association with transmission cycles restricted to specific habitats and hosts is beginning to be challenged^13–15^. Indeed, while it was believed that TcI DTU was largely predominant in Mexico and Central America (over 95% of the strains)^12,16^, recent studies have documented the presence of non-TcI parasite strains in triatomines from different regions in Mexico and Central America at high frequencies^2,14,17^. Similarly in the southern US, initial work reported only TcI and TcIV DTUs^18^, but recent studies also indicate the presence of additional non-TcI DTUs at important frequencies^19,20^. Together, these observations indicate clearly that *T. cruzi* genotype distribution in many countries and regions needs to be re-examined^16^. Also, potential parasite genetic differentiation and adaptation to specific hosts should be examined to establish the epidemiologic relevance of *T. cruzi* genotypes in the transmission of Chagas disease. As for the identification of blood feeding source mentioned above, current PCR genotyping methods are also limited to detecting the dominant genotype in biological samples, and the multiclonality of infections is mostly overlooked. Indeed, the genetic analysis of multiple parasite clones isolated from single hosts revealed concurrent infections with up to 10 parasite genotypes per opossum individual^21^. More recently, the analysis of sequence variation in GP63 through deep sequencing also revealed a large number of gene variants present in Chagasic patients^22^. The multiclonality of infection in humans is also evidenced in longitudinal studies of patients, a large proportion of which present changes in the dominant DTU identified before and after drug treatment, indicating that infection with multiple DTUs and heterogeneous drug response may be occurring^23^. Multiclonal infections may thus be the norm rather than the exception, and the interactions among parasite genotypes within vectors and hosts are still mostly unknown.

Parasite development and the vectorial capacity of vectors may also be affected by the bacteria present in the insect gut^24,25^. For example, the development of *Trypanosoma brucei*, the agent of African trypanosomiasis, in its tsetse fly vector, is directly influenced by a microbiome-regulated gut immune barrier^26^. Gut microbiome similarly modulates dengue virus infection in *Aedes aegypti* mosquitos^27,28^, and microbiome manipulation may be used to control virus transmission^29^. Triatomines harbor a diverse gut microbiome that is beginning to be identified^30,31^, but its influence on *T. cruzi* transmission remains unclear. In *Triatoma infestans*, infection by *T. cruzi* induces the overexpression of antimicrobial peptides modulating the microbiome, and this inhibition of the bacterial microflora is important for parasite establishment in the vector^32^. Nonetheless, our understanding of triatomine microbiome is still too limited to further assess its role in vectorial capacity and *T. cruzi* transmission, or to take full advantage of its potential manipulation in novel parasite control strategies such as para-transgenesis as suggested before^33,34^.

Finally, while some of the aspects mentioned above have begun to be evaluated in an isolated manner, there has been no or little effort to provide an integrative assessment of the ecological interactions that combine to shape *T. cruzi* transmission. Such interactions may however be critical for parasite transmission dynamics and Chagas disease epidemiology, and may lead to novel disease control approaches. Our objective was thus to develop an integrative and highly sensitive molecular approach using metabarcoding based on next-generation sequencing for the identification of triatomine genotype, gut microbiome, vertebrate feeding hosts, and parasite genetic diversity and their potential interactions.

## Materials and Methods

### *Triatoma dimidiata* collections and DNA extraction

A total of 14 bugs identified as *Triatoma dimidiata* were used in this study. They were collected during entomological surveillance following a pilot vector control intervention during years 2013–2015, in the villages of Bokoba, Sudzal and Teya^35^, located in the Yucatan peninsula, Mexico, about 15–20 km apart^36^, as well as in the sylvatic habitat surrounding these villages (up to 8 km from the villages)(Supplementary Table S1). The study was approved by both the World Health Organization and the Autonomous University of Yucatan institutional bioethics committees. DNA vas purified from the distal part of the abdomen of individual bugs using Qiagen DNAeasy Blood & Tissue kit following the instructions from the manufacturer, eluted in 20 µl of water, and stored at −20 °C until used.

### DNA markers amplification

We used triatomine Internal Transcribed Spacer (ITS)-2 nuclear marker for *T. dimidiata* genotyping and sibling species identification, as before^37–39^. Briefly, a 320 bp sequence was amplified (35 cycles) using the previously reported primers ITS2_200F (5′-TCGYATCTAGGCATTGTCTG-3′) and ITS2_200R (5′-CTCGCAGCTACTAAGGGAATCC-3′) and PCR conditions^40,41^.

To identify the microbiome composition, a 140 bp fragment of the bacterial 16 S rRNA gene was PCR amplified (35 cycles) using primers E786F (5′-GATTAGATACCCTGGTAG-3′) and U926R (5′-CCGTCAATTCCTTTRAGTTT-3′) as described before^42^.

For the identification of vertebrate blood meals, a 215 bp fragment of the 12 S rRNA gene was amplified (35 cycles) with the primers L1085 (5′-CCCAAACTGGGATTAGATACCC-3′ and H1259 (5′-GTTTGCTGAAGATGGCGGTA-3′) as described before^5,41,43^.

The presence of *T. cruzi* parasites in bugs was detected by PCR amplification (35 cycles) of parasite satellite DNA with primers TcZ^44,45^. Parasite genotyping was performed by multiplex PCR (40 cycles) using a mixture of three primers able to amplify *T. cruzi* DTUs TcI and TcBat (350 bp), TcII, TcV and TcVI (300 bp), and TcIV (380–400 bp), based on the mini-exon gene^46^. DNA samples purified from reference strains from the relevant *T. cruzi* DTUs were used as controls.

All PCR reactions were run using Thermo Scientific™ DreamTaq™ DNA polymerase. Positive and negative PCR controls were included in each batch of PCR reactions. A DNA extraction control using only water was also PCR amplified with each for the DNA marker used to detect potential contamination during DNA extraction. All PCR products were separated in 2% agarose gels, stained with ethidium bromide and visualized under UV light, to ensure the presence of bands of the expected size.

### Next generation sequencing

PCR products for triatomine ITS2 gene, bacterial 16 S gene, vertebrate 12 S rRNA gene, and *T. cruzi* parasite markers were pooled for each bug, and cleaned using Promega Wizard® SV Gel and PCR Clean-Up System. Following end-repair and indexing, libraries were prepared and sequenced on a MiSeq (Illumina) or Ion Torrent (Life Technologies) platforms. From 100,000 to 900,000 reads were obtained from each bug after quality and size filtering. Sequence data were deposited in the SRA database under accession number SRR6337087 to SRR6337099. Reads were aligned to reference sequences for each of the target markers in Geneious 9.1 using the Geneious algorithm, corresponding to a depth ranging from 130 to 340,000 reads per marker (Supplementary Table S1). Sequences were trimmed of primer sequences for further analysis.

### Sequence and data analysis

Sequences for bacterial 16 S gene and vertebrate 12 S rRNA gene were screened for chimeras using UChime^47^, and chimeric sequences were discarded form further analysis. Sequences for each marker were then aligned using Muscle as implemented in Geneious to assess sequence diversity. Sequence variants were identified using SNP/variant tool, to distinguish between sequencing errors/artefacts and significant sequence variants. For triatomine ITS-2 sequence analysis, variant haplotypes were aligned with reference ITS-2 sequences representing the proposed sibling species of the *T. dimidiata* complex^37,38^ for a precise taxonomic identification of the triatomines. Templeton, Crandall and Sing (TCS) haplotype networks^48^ of ITS-2 sequences were constructed in PopArt 1.7. New ITS-2 haplotype sequences were deposited in Genbank database under accession numbers: MF767417 to MF767433.

For microbiome composition, bacterial 16 S rRNA sequences were analyzed using a Bayesian classifier from the Ribosomal Database Project^49,50^ with update 5, release 11 of the sequence database. Taxonomic identification of bacteria was made at a threshold of >96% sequence identity, and the frequency of sequences for each taxonomic unit was considered as a proxy of its abundance in triatomine gut. Rarefaction curves were elaborated, at the individual and group level, to estimate species richness of our sampling. For blood meal sources, 12 S rRNA sequences were analyzed by MEGABLAST against the entire “nr” GenBank database (version of March 2017). Sequence match with >96% identity was used for species or genus identification, with an E value of at least 6.09E-77, and sequences representing less than 0.5% of a blood meal were discarded from the analysis. Rarefaction curves were also constructed. Sequences identified from the same species were further aligned to detect sequence haplotype variants as an indicator of feeding on different hosts of the same species. A feeding network and parasite transmission pathways was constructed using Cytoscape 3.5, to visualize the frequency of the respective feeding sources as well as possible pathways for parasite transmission among species when multiple blood meals were detected, since feeding sources can be used as evidence of vector-host contact. Nodes of the network represent the various species detected as feeding sources, and edges link hosts that were found in the same individual gut content. Network statistics were calculated as implemented in Cytoscape, and included the average number of neighbors, network density, network centralization, average clustering coefficient, and neighborhood connectivity distribution.

Parasite sequences from the mini-exon gene were aligned with sequences from reference strains covering all DTUs (USAOPOSSUM (TcI), Tu18 (TcII), AF1Cl7 (TcII), M6531 (TcIII), M6241 (TcIII), MT4167 (TcIV), CanIII (TcIV), SC43 (TcV), MN (TcV), CL Brener (TcVI) and VSC (TcVI)), and TCS haplotype networks were constructed using PopArt, taking into account the observed frequency of the different haplotypes within bugs, to visualize parasite diversity infecting *T. dimidiata*. Due to large differences between TcI and non-TcI sequences, these were also analyzed separately. *T. cruzi* mini-exon sequences were deposited in Genbank database under accession numbers: MF770768 to MF770822. To assess if parasites haplotypes were consistently associated within bugs, or rather circulated independently from one another, we determined which haplotypes are shared between bugs, and which are unique. This allowed identifying sets of haplotypes that can be transmitted independently from each others among vectors and hosts. Assuming that no parasite selection occurs in triatomines and a good sensitivity of our method allowing to detect rare haplotypes, this is likely to reflect independent infection events of the bugs, allowing to estimate the possible number of infection events of bugs (alternatively, it may also indicate infection on a single mammal with a multi-clonal infection).

To further describe microbiome, blood meal sources and parasite genotype sequence diversity, Shannon diversity index (H = -Σ(n_i_/N).ln(n_i_/N)) as well as Margalef Richness index ((S-1)/ln N) were calculated, with n_i_ representing the number of individuals of species/taxa i, N the total number of individuals, and S the total number of species/taxa. Indices were compared using Student’s t test, and linear regression was used to assess the potential associations among biodiversity indicators. Frequency data were compared by χ^2^ or Fisher’s Exact tests.

## Results

### Triatoma dimidiata ITS-2

Over 260,000 partial ITS-2 sequences (209 bp long) were analyzed, corresponding to an average of 18,627 sequences per bug (N = 14). Four bugs presented a single ITS-2 sequence, and eight presented two sequence haplotypes at a frequency of about 50% each, suggesting that these corresponded to heterozygote bugs. Interestingly, one bug presented four haplotypes, at frequencies of 46, 39, 9 and 6%, respectively. Two of these four sequences were novel and not found in other bugs, thus were not a contamination from other samples, but we could not exclude that they may represent chimeric sequences. Overall, these data confirmed the extensive concerted evolution of this multi-copy gene, which mostly behaves as a single copy sequence. It also confirmed the filtering out of errors/artefacts throughout our amplification, sequencing and analysis process.

**Figure 1:**
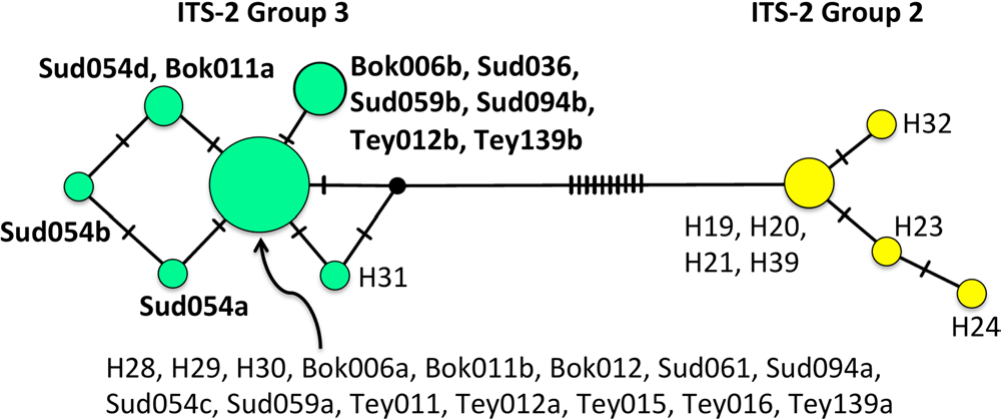
ITS-2 haplotypes of *T. dimidiata*. Partial ITS-2 sequences were aligned with reference haplotypes H19, H20, H21, H23, H24, H28, H29, H30, H31, H32, covering Groups 2 and 3 from Genbank, and a TCS network was constructed in PopArt. Circles represent the respective haplotypes, their size proportional to the number of sequences. Ticks on branches indicate the number of mutations separating haplotypes. ITS-2 Group 2 (yellow) and Group 3 (green) are clearly separated by at least 11 mutations as described before. Haplotypes labeled in bold font correspond to new haplotypes.

Comparison of ITS-2 haplotypes with reference haplotypes confirmed that all analyzed bugs belonged to the previously described Group 3 of this species complex^37,38^, and the haplotype of most bugs corresponded to known ITS-2 haplotypes (Fig. 1). Eleven bugs (Bok006, Bok011, Bok012, Sud059, Sud061, Sud094, Tey011, Tey012, Tey015, Tey016, Tey139) presented haplotypes identical to H28, H29 and H30. Four new haplotypes were identified differing in 1 nucleotide each: Sud036, also observed in three other bugs (Sud059, Sud094, and Tey139), and three additional haplotypes were all observed in bug Sud054. There was no association between the ITS-2 haplotype and the village of collection of the bugs (Fisher’s Exact Test, *P* = 0.8368).

### Microbiome composition

**Figure 2:**
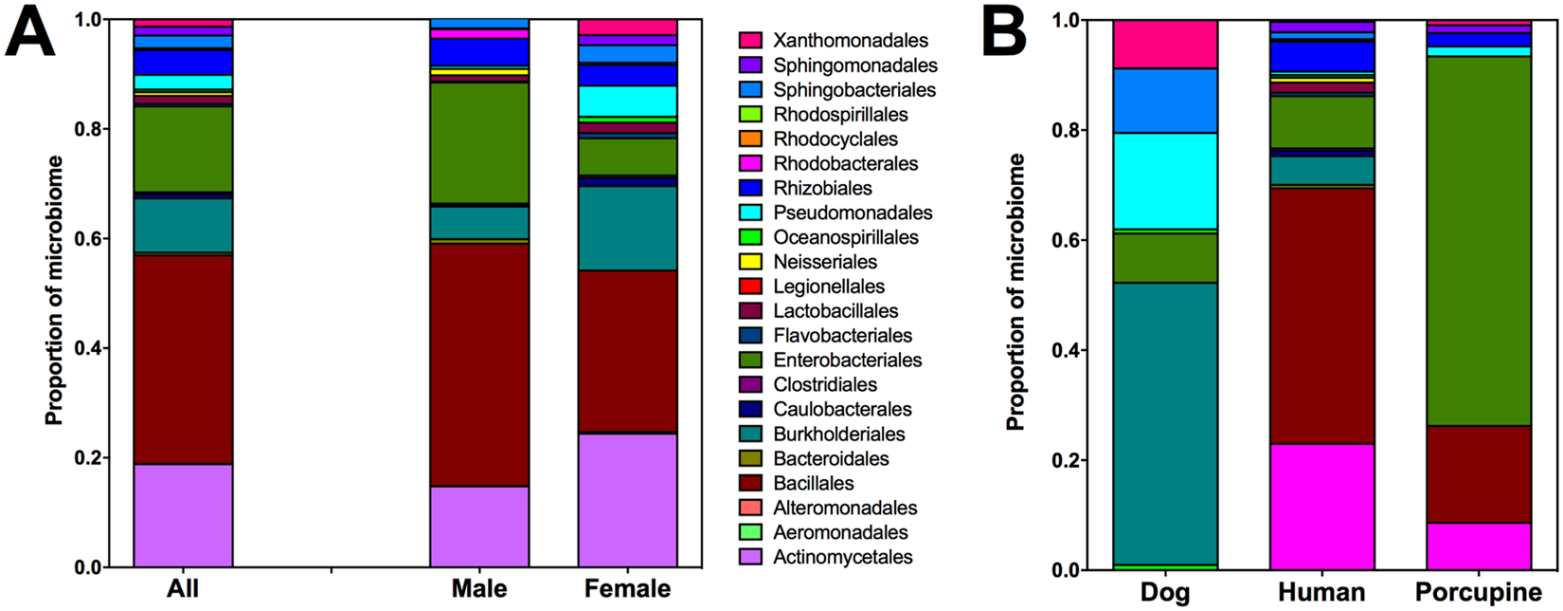
Gut microbiome composition of *Triatoma dimidiata*. The average composition of the microbiome from 14 individuals is shown, to the level of bacterial order (**A**). There are significant differences between male and female microbiomes, with females presenting a greater diversity of orders. (**B**) Microbiome composition is also significantly different depending on the dominant blood meal present in triatomine gut, which was identified by analysis of 12 S rARN vertebrate sequences (see below).

About 500,000 partial 16 S rRNA sequences (110 bp long) were analyzed, corresponding to an average of 37,654 sequences per bug (N = 13). A total of 23 bacterial orders were identified with high confidence, and rarefaction curve indicated that most of the richness had been identified through our sampling (Supplementary Figure S1), although some additional low abundance taxa may remain to be detected. The overall Shannon diversity index (H) was 1.41 and Margalef richness index was 1.90. The most abundant orders were Bacillales, followed by Actinomycetales, Enterobacteriales and Burkholderiales, which accounted for over 70% of *T. dimidiata* microbiome (Fig. 2A), and are commonly found in other insect gut microbiome including triatomines^31,51,52^. The main bacteria genus identified were *Rhodococcus, Corynebacterium, Actinomycetospora* and *Arthrobacter* (from the Actnomycetales order) and *Bacillus, Staphylococcus* and *Anoxybacillus* (from the Bacillales order). Importantly, *Wolbachia* was not detected in any of the samples, indicating that it might be absent from this insect species.

**Figure 3:**
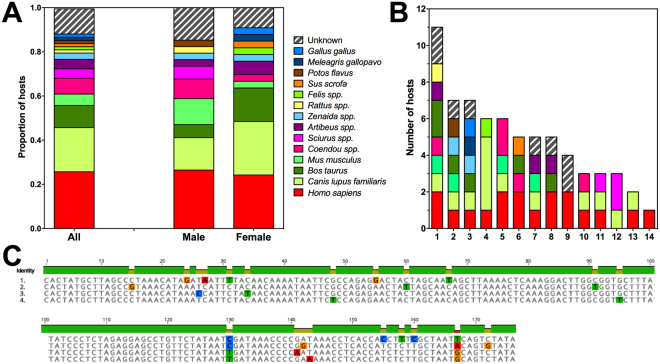
Blood feeding sources of *Triatoma dimidiata*. The sources of blood were identified to the genus or species level by analysis of 12 s rRNA sequences obtained by NGS, and corresponded to 14 genus/species (**A**). Females tended to feed on a wider diversity of species, but this did not reach significance. (**B**) Feeding on multiple species or individuals was detected in most individual bugs (bugs 1–14), with an average of 4.9 ± 0.7 hosts/bug. (**C**) Multiple dog 12 S haplotypes detected in a single bug (Bok012), which had feed on at least 4 dogs. Nucleotides highlighted in color indicate sequence variants. These data indicate very frequent host-switching when feeding, within and among species.

Interestingly, the composition of bacterial orders was significantly different between males and females (χ^2^ = 141.3, d.f. = 18, *P* < 0.0001), with females presenting a larger diversity of orders in their microbiome (Fig. 3A). Margalef Richness index was indeed higher in females compared to males (1.49 ± 0.16 vs 0.97 ± 0.21, respectively, *P* = 0.041), and Shannon Index tended to be higher in females (1.70 ± 0.14 vs 1.40 ± 0.29, *P* = 0.19). Oceanospirillales, Pseudomonadales, Xanthomonadales, Flavobacteriales, Caulobacterales, Burkholderiales and Actinomycetales were more abundant in females, while Bacillales, Bacteroidales, Enterobacteriales, Neisseriales and Rhodobacterales were more abundant in males. More strikingly, microbiome composition differed strongly based on the dominant blood meal source of the bugs (Fig. 2B), with Burkholderiales predominating in bugs fed on dogs, Bacillales in those fed on humans, and Enterobacteriales for those fed on porcupine, respectively (χ^2^ = 519.5, d.f. = 36, *P* < 0.0001).

### Vertebrate host feeding

About 218,000 partial vertebrate 12 S rRNA sequences (about 170 bp long) were analyzed for the identification of blood sources of triatomines, corresponding to an average of 15,573 sequences per bug (N = 14). MEGABLAST analysis revealed a diverse range of at least 14 host species, including the expected domestic and synantropic species such as dog (*Canis lupus familiaris*), cow (*Bos taurus*), cat (*Felis spp*.), mouse (*Mus musculus*), rat (*Rattus* spp.), pig (*Sus scrofa*), turkey (*Meleagris gallopavo*) and chicken (*Gallus gallus*), in addition to humans (*Homo sapiens*) (Supplementary Table 2 for BLAST scores). Sylvatic species were also detected, corresponding to porcupine (*Coendou* spp.), squirrel (*Sciurus* spp.), fruit bat (*Artibeus* spp.), kinkajou (*Potos flavus*), and dove (*Zenaida* spp.) (Supplementary Table 2 for BLAST scores), with a small proportion of sequences with ambiguous or unidentified origin (Fig. 3A). Only four species (human, dog, cow and mice) accounted for over 50% of blood sources, and may thus play a key role in parasite transmission cycles. There were small differences in blood sources of male and female triatomines, with a tendency of females to have a somewhat wider range of hosts (Fig. 3A), but this did not reach statistical significance (χ^2^ = 12.5, d.f. = 14*, P* = 0.56). Shannon diversity index for the blood sources of female and male bugs was also similar (0.44 ± 0.18 vs 0.39 ± 0.16, respectively, *P* = 0.43), as well as the Margalef richness index (0.43 ± 0.18 vs 0.34 ± 0.10, respectively, *P* = 0.33).

At the level of individual bugs, variations in sequence frequency allowed to easily identify a dominant (and likely last) blood meal, accounting for 50–100% of the sequences, while lower frequency sequences may correspond to older or partial blood meals. Thus, multiple host species were detected in all bugs except one, with up to 7 identified host species in a single bug (Fig. 3B). Within species, multiple haplotypes were also detected (Fig. 3C), so that the average number of hosts/bug reached 4.9 ± 0.7. Based on the persistence of blood DNA in triatomine gut for about 5–6 weeks after a blood meal^53^, these data indicate an average of about 50 hosts/year for each bug, up to about 115 hosts/year. These results also show frequent feeding on different hosts by individual bugs, which suggested an opportunist rather than specialized feeding behavior of *T. dimidiata*. The mixture of domestic and sylvatic species as blood sources among several individual bugs further confirmed the occurrence of widely overlapping parasite transmission cycles among species.

**Figure 4:**
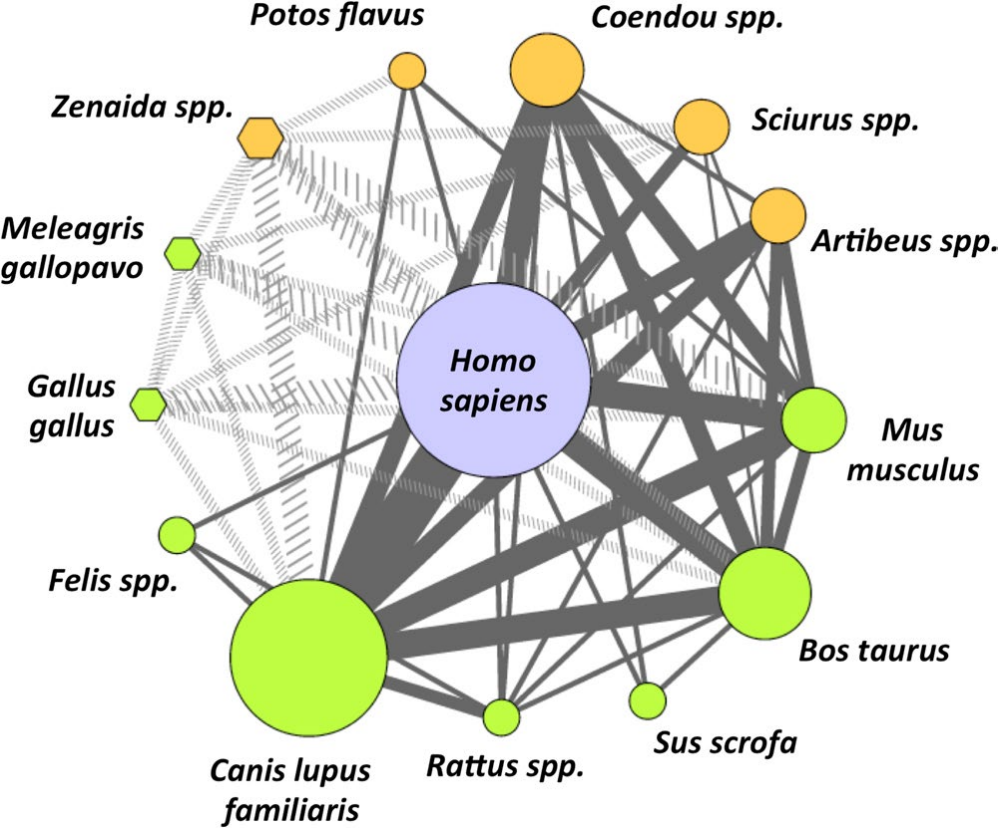
Feeding and possible parasite transmission network of *T. dimidiata*. Blood source nodes correspond to domestic (green symbols) and sylvatic (orange symbols) host species, as well as humans (blue), with the size proportional to the feeding frequency on each host. Diamond shaped nodes represent birds, which do not carry *T. cruzi* parasites, and circles represent mammals, which can be infected by *T. cruzi*. Edges link species which are found together in multiple blood meals within individual bugs, and the width of the lines is proportional to the frequency of the association between species. Solid dark gray lines link mammalian species, among which *T. cruzi* may circulate, while dotted light gray lines involve bird species, which only serve as blood sources for the bugs. Humans may thus become infected by *T. cruzi* parasites originating from dogs, cows and mice, as well as from sylvatic hosts such as porcupines, squirrels and fruit bats. Dogs can play a key role as domestic host/reservoir favoring parasite transmission to humans. On the other hand, cats, rats and pigs play a secondary role in parasite transmission.

To further assess potential transmission cycles of *T. cruzi* parasites by *T. dimidiata* among vertebrate host species, a feeding and transmission network was constructed (Fig. 4). Feeding frequency on each host is indicated by the size of the node, to reflect the more common feeding sources, while connecting edges between species are based on the observed occurrence of multiple feeding sources within single bugs. Since birds cannot carry *T. cruzi* parasites, they only play a role as blood source for triatomines, which is indicated by dotted edge connections between hosts, the solid lines between mammals indicate potential parasite transmission pathways. The observed network shows a strong connectivity (average number of neighbors: 7.57, network density 0.58) and strong clustering (average clustering coefficient: 0.85) with a medium centralization (network centralization: 0.49) (Fig. 4), which illustrates the frequent host-switching behavior of *T. dimidiata*. Analysis of neighborhood connectivity distribution showed that neighborhood connectivity decreased strongly with the number of neighbors (Supplementary Figure S2, R2 = 0.96, *P < *0.0001), indicating that highly connected nodes predominate in shaping the network. Indeed, the network shows the limited role of birds as feeding sources, while a mixture of domestic and sylvatic mammalian species contribute to potential parasite transmission pathways (Fig. 4). Humans may thus become infected by *T. cruzi* parasites originating from dogs, cows and mice, as well as from sylvatic hosts such as porcupines, squirrels and fruit bats. Particularly, dogs can play a key role as domestic host/reservoir favoring parasite transmission to humans, while, cats, rats and pigs play a secondary role in parasite transmission.

### *Trypanosoma cruzi* infection

**Figure 5:**
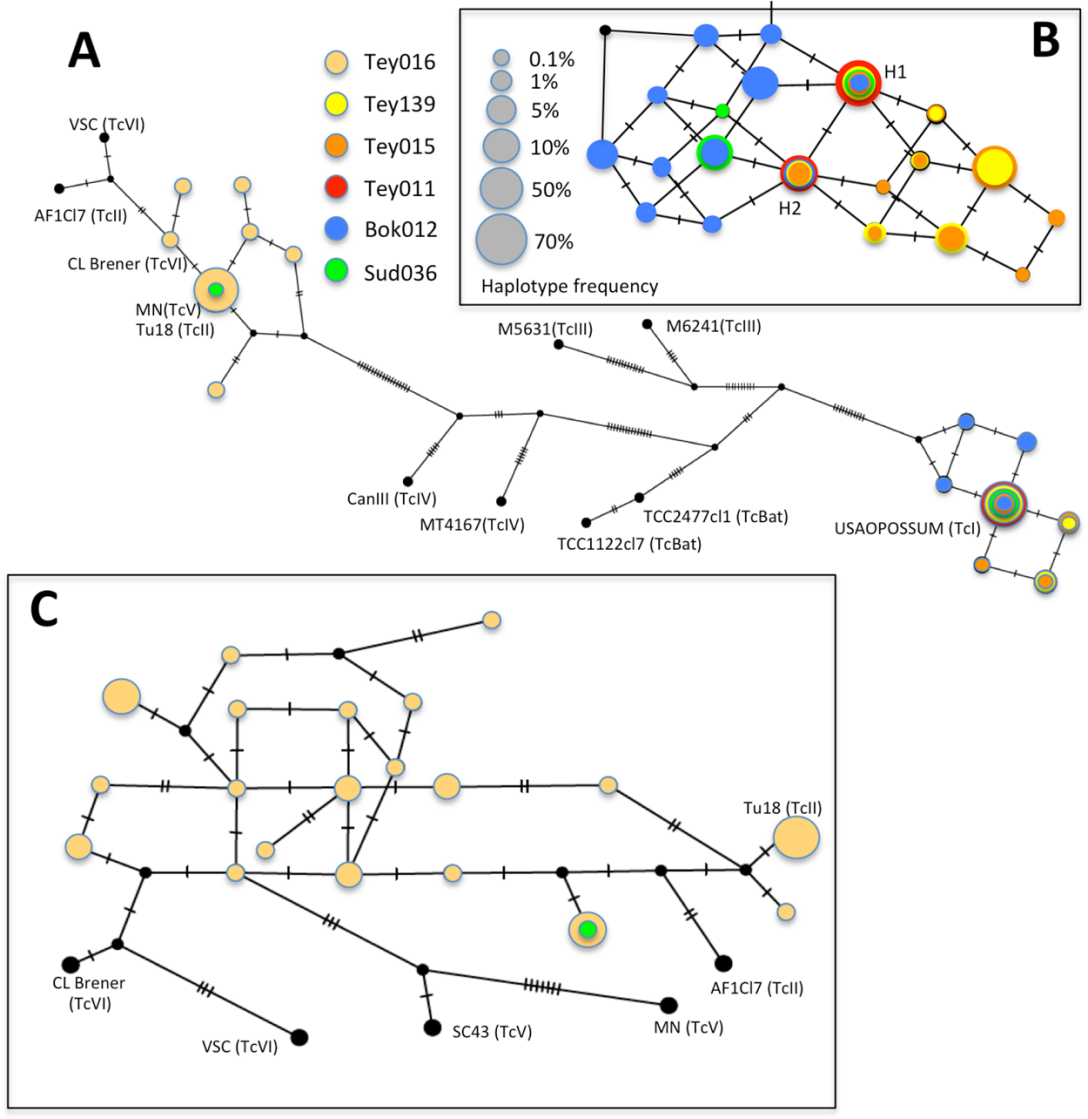
*Trypanosoma cruzi* genetic structure in *Triatoma dimidiata*. TCS network were constructed based on sequences from the mini-exon gene. Circles represent unique *T. cruzi* haplotypes, with the size of the circle proportional to their frequency, and the colors correspond to the indicated bugs harboring the parasites. Tey000 are bugs from the village of Teya, while Sud036 is from the village of Sudzal, and Bok012 from the village of Bokoba. Ticks on branches between haplotypes indicate the number of nucleotide substitutions separating each haplotypes. (**A**) Complete network shows the diversity of parasite DTUs present, including TcI and TcII-V-VI DTUs, which are not resolved in this global analysis. (**B**) Detailed network of TcI haplotypes, showing that some haplotypes are found in most bugs (H1 and H2), while others are found in only a few or even a single bug. The independent occurrence of haplotypes in different bugs, and at variable frequencies, suggest the occurrence of multiple infection events. (**C**) Detailed network analysis of TcII, TcV and TcVI DTU sequences.

About 50,000 partial mini-exon sequences (260–300 bp long) from *T. cruzi* were analyzed from infected *T. dimidiata* bugs for the characterization of parasite diversity in triatomines, corresponding to an average of 8,420 sequences per bug (N = 6). This allowed the detection of an important haplotype diversity within each individual triatomine, ranging from 2 to 20 parasite haplotypes, with frequencies ranging from 0.1% to 65% (Tables 1 and 2). TCS network analysis indicated that haplotype diversity covered several DTUs, including TcI, as well as TcII-TcV-TcVI DTUs, but TcIII, TcIV and TcBat were not detected (Fig. 5A). For a finer resolution, TcI and non-TcI sequences were thus analyzed separately. For TcI, a total of 20 haplotypes were detected, with up to 12 in a single bug (Bok012), and only 2 in Tey011 bug (Fig. 5B). For TcII, TcV and TcVI, 20 haplotypes were detected, all also present in a single bug (Tey016). Several of these haplotypes were identical or clearly related to TcII DTU, and others were somewhat closer to TcV and TcVI reference sequences, but could not be easily assigned to a specific DTU. One bug (Sud036) harbored haplotypes from three TcI and one TcII DTUs. Some haplotypes were shared among bugs (for example TcI-H1 and H2 shared by 5 and 4 bugs, respectively), while others were more exclusive, suggesting the independent circulation of many groups of haplotypes among bugs (Fig. 5B,C). While the mini-exon gene is a multi-copy sequence^54^, which may thus present some level of paralogous sequence variation within a single parasite clone/genome, the detection of independent groups of haplotypes circulating among bugs and of multiple parasite DTUs strongly support at least some level of multiclonality of *T. cruzi* infection in *T. dimidiata*. Based on the independent groups of haplotypes, the number of likely infection events could be estimated for individual bugs, and up to 4 infection events were detected in some cases.

**Table 1:** *Trypanosoma cruzi* haplotypes of the mini-exon (TcI DTU) and their frequencies in *T. dimidiata*

**Bug ID or reference ID**	**Haplotype-DTU**	**Freq. (%)**	**Position in sequence**								**Genbank accession**
			**9**	**16**	**20**	**126**	**192**	**195**	**215**	**234**	
USAOPOSSUM	TcI	NA	A	T	G	T	C	C	T	C	JQ581510
Bok012	H1-TcI	24.4	—	—	—	—	—	—	C	—	MF770768
Bok012	H2-TcI	19.8	—	C	—	—	—	—	C	—	MF770769
Bok012	H3-TcI	16.6	—	C	T	C	—	—	C	—	MF770770
Bok012	H4-TcI	10.4	—	C	—	—	—	—	—	—	MF770771
Bok012	H5-TcI	8.3	—	—	—	C	—	—	C	—	MF770772
Bok012	H6-TcI	6.5	—	—	—	—	—	—	—	—	MF770773
Bok012	H7-TcI	5.2	—	C	T	—	—	—	C	—	MF770774
Bok012	H8-TcI	2.4	—	C	T	C	—	—	—	—	MF770775
Bok012	H9-TcI	2.1	—	C	—	C	—	—	C	—	MF770776
Bok012	H10-TcI	1.8	—	C	T	—	—	—	—	—	MF770777
Bok012	H11-TcI	1.6	—	—	—	C	—	—	—	—	MF770778
Bok012	H12-TcI	1.0	—	C	—	C	—	—	—	—	MF770779
Sud036	H2-TcI	55.8	—	C	—	—	—	—	C	—	MF770782
Sud036	H13-TcI	23.2	—	—	—	—	—	—	—	T	MF770783
Sud036	H1-TcI	21.0	—	—	—	—	—	—	—	—	MF770781
Tey011	H1-TcI	64.6	—	—	—	—	—	—	—	—	MF770784
Tey011	H14-TcI	35.4	—	C	—	—	—	—	—	—	MF770785
Tey015	H15-TcI	40.2	—	—	—	—	G	T	—	—	MF770788
Tey015	H16-TcI	33.9	—	C	—	—	G	T	—	—	MF770789
Tey015	H1-TcI	17.2	—	—	—	—	—	—	—	—	MF770786
Tey015	H14-TcI	7.0	—	C	—	—	—	—	—	—	MF770787
Tey015	H17-TcI	1.2	C	—	—	—	G	T	—	—	MF770790
Tey015	H18-TcI	0.2	C	C	—	—	G	T	—	—	MF770791
Tey015	H19-TcI	0.1	—	—	—	—	G	—	—	—	MF770792
Tey015	H20-TcI	0.1	—	—	—	—	—	T	—	—	MF770793
Tey015	H21-TcI	0.1	—	C	—	—	—	T	—	—	MF770794
Tey015	H22-TcI	<0.1	—	C	—	—	G	—	—	—	MF770795
Tey139	H15-TcI	38.9	—	—	—	—	G	T	—	—	MF770818
Tey139	H16-TcI	33.1	—	C	—	—	G	T	—	—	MF770819
Tey139	H1-TcI	15.5	—	—	—	—	—	—	—	—	MF770816
Tey139	H14-TcI	7.9	—	C	—	—	—	—	—	—	MF770817
Tey139	H19-TcI	0.1	—	—	—	—	G	—	—	—	MF770820
Tey139	H20-TcI	0.1	—	—	—	—	—	T	—	—	MF770821
Tey139	H22-TcI	0.1	—	C	—	—	G	—	—	—	MF770722

NA: not applicable. Freq.: frequency.

**Table 2:** *Trypanosoma cruzi* haplotypes of the mini-exon (non-TcI DTU) and their frequencies in *T. dimidiata*.

**Bug ID or ref ID**	**Haplotype-DTU**	**Freq. (%)**	**Position**																				**Accession**
			**70**	**72**	**73**	**76**	**77**	**78**	**79**	**87**	**88**	**94**	**113**	**121**	**122**	**124**	**126**	**159**	**193**	**245**	**252**	**263**	
Tu18	TcII	NA	G	G	C	C	A	C	C	T	C	T	C	T	A	G	C	T	A	T	G	G	AY367125
VSC	TcVI	NA	T	—	T	T	G	T	G	C	G	A	T	—	—	C	—	C	—	del	del	T	FJ463159
MN	TcV	NA	T	C	T	T	G	T	G	C	G	—	—	—	—	—	T	—	—	—	—	—	AY367128
Sud036	H42-NoTcI	0.1	—	—	—	—	—	—	—	C	G	—	—	—	G	T	—	—	—	—	—	—	MF770780
Tey016	H23-NoTcI	19.8	—	—	—	—	—	—	—	—	—	—	—	—	G	T	—	—	—	—	—	—	MF770797
Tey016	H42-NoTcI	14.2	—	—	—	—	—	—	—	C	G	—	—	—	G	T	—	—	—	—	—	—	MF770796
Tey016	H24-NoTcI	12.0	T	—	T	—	—	—	—	—	—	—	T	—	—	—	—	C	—	—	—	A	MF770798
Tey016	H25-NoTcI	6.4	T	—	T	T	G	T	G	—	—	A	T	—	—	—	—	—	G	—	—	—	MF770799
Tey016	H26-NoTcI	6.1	T	—	T	—	—	—	—	C	G	—	T	—	—	—	—	—	—	—	—	—	MF770800
Tey016	H27-NoTcI	6.1	T	—	T	—	—	—	—	C	G	—	T	—	—	—	—	—	—	—	—	A	MF770801
Tey016	H28-NoTcI	6.1	T	—	T	—	—	—	—	C	G	—	—	—	—	—	—	—	—	—	—	A	MF770802
Tey016	H29-NoTcI	4.5	T	—	T	T	G	T	G	T	C	—	T	C	—	—	—	—	G	—	—	—	MF770803
Tey016	H30-NoTcI	3.9	T	—	T	—	—	—	—	C	G	—	T	C	—	—	—	—	—	—	—	A	MF770804
Tey016	H31-NoTcI	3.1	T	—	T	—	—	—	—	C	G	—	T	C	—	—	—	—	—	—	—	—	MF770805
Tey016	H32-NoTcI	3.1	T	—	T	T	G	T	G	—	—	—	T	—	—	—	—	—	—	—	—	—	MF770806
Tey016	H33-NoTcI	3.1	T	—	T	T	G	T	G	—	—	A	T	—	—	—	—	—	G	—	—	A	MF770807
Tey016	H34-NoTcI	1.9	—	—	—	—	—	—	—	C	G	—	—	—	—	—	—	—	—	—	—	A	MF770808
Tey016	H35-NoTcI	1.7	—	—	T	—	—	—	—	C	G	—	T	—	—	—	—	—	—	—	—	—	MF770809
Tey016	H36-NoTcI	1.7	T	—	T	T	G	T	G	—	—	—	T	—	—	—	—	—	—	—	—	A	MF770810
Tey016	H37-NoTcI	1.4	T	—	T	T	G	T	G	—	—	—	T	C	—	—	—	—	—	—	—	A	MF770811
Tey016	H38-NoTcI	1.1	T	—	T	—	—	—	—	C	G	—	T	C	—	—	—	—	G	—	—	—	MF770812
Tey016	H39-NoTcI	1.1	—	—	—	—	—	—	—	—	—	—	T	—	G	T	—	—	—	—	—	—	MF770813
Tey016	H40-NoTcI	1.1	T	—	T	T	G	T	G	T	C	—	T	—	—	—	—	—	—	—	—	A	MF770814
Tey016	H41-NoTcI	0.1	T	—	T	—	—	—	—	C	G	—	T	—	—	—	—	C	—	—	—	A	MF770815

NA: not applicable, NoTcI: non-TcI DTU, del: deletion.

**Figure 6:**
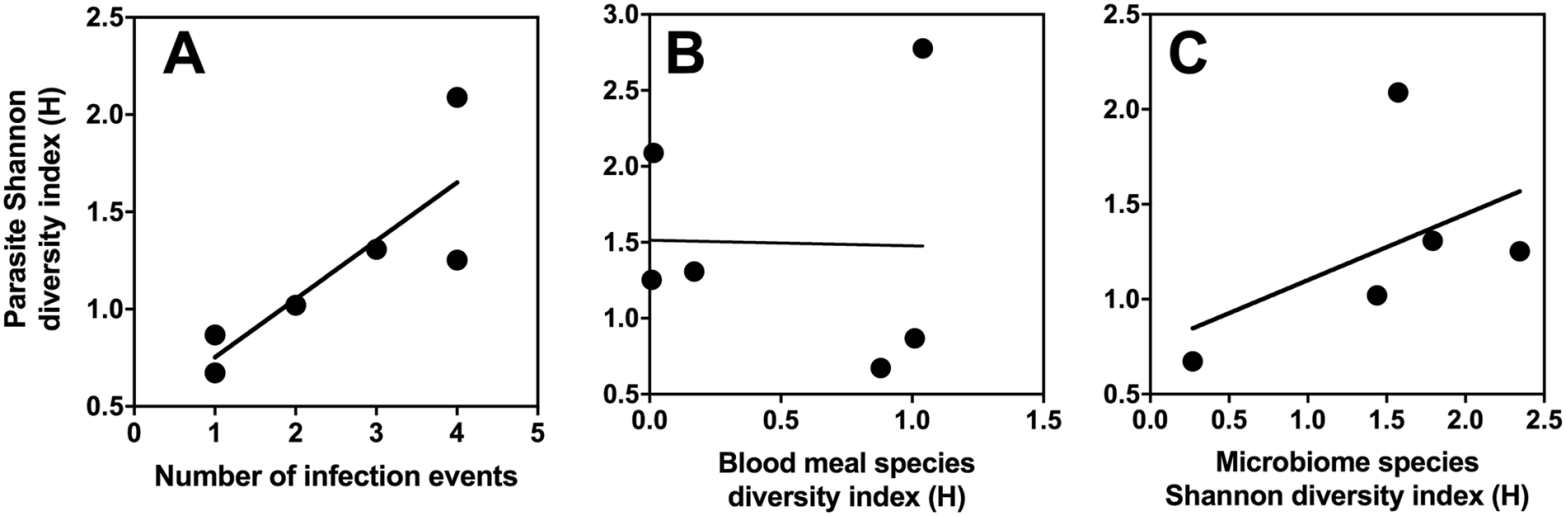
Association between parasite diversity, feeding sources and microbiome diversity. Shannon diversity index was used to compare the overall diversity of *T. cruzi* parasite, feeding sources and gut microbiome diversity in *T. dimidiata* bugs. (**A**) Parasite diversity significantly increased with the number of infection events (R^2^ = 0.69, *P* = 0.039). (**B**) There was no association between parasite diversity and blood meal species diversity (*P* = 0.96). (**C**) Parasite diversity tended to increase with microbiome diversity, but this did not reach statistical significance (R^2^ = 0.25, *P* = 0.38).

Shannon diversity index was then used to compare the diversity of *T. cruzi* parasites with the number of infection events, feeding sources and gut microbiome diversity in individual *T. dimidiata* bugs (Fig. 6). As expected, parasite diversity significantly increased with the number of infection events (R^2^ = 0.69, *P* = 0.039). Unexpectedly, we could not detect an association between parasite diversity and blood meal species diversity (*P* = 0.96), possibly because of our small sample size combined with the fact that only recent blood meals can be detected, while bugs remain infected with *T. cruzi* for their lifespan. Also, parasite diversity tended to increase with microbiome diversity, but this did not reached statistical significance (R^2^ = 0.25, *P* = 0.38).

## Discussion

Molecular approaches are providing very relevant information on *T. cruzi* transmission and the risk for human infection in many settings. However, most of these approaches have limited sensitivity to assess the full diversity of triatomine feeding hosts and parasite genotypes, or lack integration. We developed here a metabarcoding strategy based on next-generation sequencing to successfully identify a wide range of ecological associations of *T. dimidiata* in the Yucatan peninsula, Mexico. Analysis of triatomine ITS-2 sequences confirmed that our study focused on ITS-2 Group 3 of *T. dimidiata* species complex. This group is clearly differentiated from the other ITS-2 group^37,38^, with a different ecological niche at the regional level^55^, although it appears to have a comparable epidemiological role as other sub-species from the *dimidiata* complex^39,56,57^. The lack of ITS-2 sequence variants in most bugs also confirmed the strong concerted evolution of this multi-copy marker, as well as the reliability of our methodological approach.

We also obtained extensive information on *T. dimidiata* gut microbiome for the first time. We identified that the most common bacterial orders present are similar to those found in the microbiome of other insect vectors such as *Aedes*, as well as other triatomine species. However, there were also some differences among *T. dimidiata* and other triatomines. For example, Clostridiales and Rhodocyclales are more frequent in *Triatoma infestans, Rhodnius prolixus*, and *Panstrongylus megistus* but marginally present in *T. dimidiata*^51^. Enterobateriales predominate in *Rhodnius prolixus*^52^, while Bacillales are more abundant in *T. dimidiata*. Rhizobiales, Burkholderiales, Sphingomonadales, Pseudomonadales, Caulobacterales, Lactobacilales and Xanthomonadales are also relatively abundant in *T. dimidiata*, but have not been reported in other triatomines^51^, although they have been observed in mosquitoes such as *Aedes* spp., *Culex* spp.^58–60^ or ants^61^. Remarkably, *Wolbachia* was not detected in *T. dimidiata*, in agreement with its absence from triatomine species such as *Triatoma infestans, Triatoma vitticeps, Dipetalogaster maximus, Panstrongylus maximus*^30^, *Triatoma brasiliensis* and *Triatoma pseudomaculata*^31^, and *Rhodnius* remains the only triatomine genus in which *Wolbachia* has been detected in both natural and laboratory populations^62^.

*T. dimidiata* microbiome appeared as very dynamic, with differences between males and females, which may reflect differences in nutrient needs. Blood feeding sources may also affect microbiome composition, as suggested by our observation of differing microbiomes among bugs with different dominant blood sources, and further studies are needed to clarify this point. In mosquitoes, the microbiome has been found to be similarly very dynamic, showing seasonal variations^63,64^, as well as changes due to the environment, diet, and aging^65^, although a constant core microbiome could be identified. Further studies should help clarify the variations in *T. dimidiata* microbiome composition according to its ecological conditions.

Blood feeding was observed on at least 14 host species, most of which had been reported before as feeding sources for *T. dimidiata*^2,6,66–68^, with the exception of some of the sylvatic hosts such as the kinkajou, porcupine, squirrel and fruit bat. However, the most striking observation was that all bugs except one had blood from more than one host, with up to 11 hosts detected in a single bug. This is considerably higher that what has been observed with previous molecular approaches, which at best can detect 3–5 hosts/bug^4–6^, and most studies report unmixed/single blood meals^7^. This highlights the high sensitivity of our deep sequencing approach. We were thus able to identify not only the dominant blood meal, likely corresponding to the most recent/abundant meal, but also the remaining older or partial blood meals present in much lower amount. The identification of blood from different hosts in a bug represents key evidence of host-switching, which appears to be very frequent for *T. dimidiata*. Feeding frequency of triatomines has been a long sought parameter, as it contributes to transmission dynamics^69^. Estimates from field studies suggested that it was around a blood meal every 1.7–7 days for *T. infestans*^7,70^. Our data suggest about one (at least partial) blood meal every week, up to one every 3 days, which is very consistent with these previous studies. We thus estimated that *T. dimidiata* could feed on up to 115 hosts per year, which may provide many opportunities for parasite circulation among hosts and individuals and/or species. Such a large number of feeding hosts is also considerably higher than for other vector species such as mosquitoes, which at best feed on a handful of hosts during their lifespan^69^, and this is likely to strongly affect parasite transmission dynamics^71^. In addition, the mixture of blood meals on domestic and sylvatic hosts confirmed the strong overlap of domestic and sylvatic transmission cycles, which may reflect a high mobility of both vectors and hosts between these habitats. This is in agreement with the intrusive behavior of *T. dimidiata* in this region^72–77^.

Information on multiple blood meals was used to construct potential *T. cruzi* transmission network as it evidences chains of vector-host contacts. Dogs emerged as a key host for parasite transmission to humans, as observed in other regions^78–80^, and also in agreement with a relatively high prevalence of infection of this host^81,82^. This observation strengthens the rationale for controlling *T. cruzi* infection in dogs as part of an integral control intervention. This may be achieved by insecticide treatment^83–85^ or a vaccine^86,87^. Mice also seem to play an important role in parasite transmission, although their infection rate by *T. cruzi* may be highly variable in the region^81,88,89^. On the other hand, our network indicated that rats and cats played a limited role in parasite transmission to humans, in spite of a significant prevalence of *T. cruzi* infection observed in previous studies^81,90^. Nonetheless, rodent control has been successfully tested as part of a Chagas disease control intervention in Guatemala^91^. Cows rather than pigs are other domestic animals that appear to contribute to the circulation of *T. cruzi* in our setting, although limited information is available about *T. cruzi* infection in these hosts^92^. Importantly, several sylvatic host species including porcupine, squirrel and fruit bat, reported here for the first time as important blood sources for *T. dimidiata*, were also strongly connected to potential parasite transmission to humans and other domestic hosts, emphasizing the direct links between sylvatic and domestic hosts of *T. cruzi*. Importantly, fruit bats have previously been found to be infected by *T. cruzi* (about 3% for *Artibeus* spp.) in the southern part of the Yucatan peninsula^89^.

Unexpectedly, no blood meals were detected from *Didelphis* opossums, which have been reported with a very high *T. cruzi* infection rate in the region (12–54%)^81,89,93^. This may be due to the limited number of bugs analyzed, but infection through other routes such as oral or through anal gland may also play a role for these hosts. PCR primer bias may also occur, although we successfully amplified control opossum DNA from Yucatan with our primers, and opossum has been previously reported using the same primers^6^. Analysis of additional populations of *T. dimidiata*, and of the seasonal variations in feeding profiles using our approach should provide additional information to refine feeding and transmission networks, and of the potential source of parasites infecting humans.

Analysis of *T. cruzi* genetic diversity revealed a very high level of parasite diversity in *T. dimidiata*, with up to 20 parasite haplotypes per bug, based on the analysis of the mini-exon marker. While this diversity may be due to sequence variants in paralogous copies within clones/genomes of this repeated sequence, variations in haplotype frequencies, their apparent independent circulation among bugs, and the presence of several well established parasite DTUs all support extensive multiclonality of infection. While most studies focus on identifying the main dominant genotype circulating in vectors and overlook this multiclonality^14,89,94–96^, our data emphasize the very high sensitivity of our approach, which provided detailed information on the composition of parasite populations infecting bugs, not only at the level of sequence haplotype, but also on their relative frequency. Indeed, we were able to detect even very low frequency haplotypes, representing as little as 0.1% of the parasite haplotypes present in *T. dimidiata*. Some of this diversity may be due to contamination among samples following incorrect indexing, which would tend to minimize differences among bugs, or may also represent chimeric sequences and/or sequencing artefacts. However, we also detected rare parasite haplotypes in a single bug (for example H18-TcI (0.2%) is unique to Tey015, or H41-NoTcI (0.1%) is unique to Tey016), which are more likely to be true sequence variants.

A high clonal diversity of *T. cruzi* in *T. dimidiata* is in agreement with the extensive host switching behavior described above, as bugs feeding on a large variety of hosts may likely get infected with a large variety of parasites. Using Shannon diversity index as an indicator of parasite diversity, we could confirm the significant correlation between parasite genetic diversity with the number of infection event of bugs. However, we could not detect any correlation between blood sources diversity and parasite diversity. As mentioned above, this may be due to small sample size, combined with the fact that blood meals only reflect recent feeding host, while *T. cruzi* infection lasts for the entire lifespan of the bugs. On the other hand, parasite diversity may be associated with microbiome diversity, and future studies should help establish the critical interactions between triatomine microbiome and *T. cruzi* development^51^.

TcII DTU was detected in *T. dimidiata* for the first time in the region, and possibly additional non-TcI DTUs are also present since our analysis revealed a large number of haplotype variant within the closely related TcV and TcVI DTUs. Although the reference sequences from TcII, TcV and TcVI DTUs are clearly separated, we observed many intermediate sequences much harder to classify. Further studies using additional markers should help refine parasite genetic structure, and the population dynamics resulting from interactions among parasite genotypes. Overall, our results are in agreement with the large diversity of *T. cruzi* strains and DTUs observed in *T. dimidiata* in Veracruz, Mexico^13^, and Guatemala^17^, as well as in vertebrate hosts from the Yucatan^89^, even though these studies only documented the dominant genotypes as mentioned above. Our observations raise the questions of the nature of the interactions among parasite haplotypes within vectors, which may play an important role in shaping parasite transmission and the epidemiology of *T. cruzi* infection^97,98^. In any case, it is clear that future studies should take into account the multiclonality of *T. cruzi* infections to further understand parasite transmission networks and the epidemiology of Chagas disease.

In conclusion, we described here a powerful new approach based on metabarcoding through next generation sequencing to provide detailed information on ecological associations of *T. dimidiata* bugs in an integrative manner. Importantly, our analysis provided much greater sensitivity than conventional molecular approaches to detect multiple blood meals in bugs as well as multiclonal infections with *T. cruzi*. Moreover, our observations confirm the presence of a very dynamic triatomine microbiome, which may be modulated by blood sources and may interact with *T. cruzi* parasite. This extensive diversity of feeding sources and parasite infections allowed documenting transmission network. Expanding these studies to additional *T. dimidiata* populations and at different times of the year will provide unprecedented data to characterize *T. cruzi* parasite transmission and the risk for human infection, and which ultimately will lead to a better control of disease transmission.

## Electronic supplementary material

Supplementary materials
