# Supplementary material for: Detailed ecological associations of triatomines revealed by metabarcoding and next-generation sequencing: implications for triatomine behavior and Trypanosoma cruzi transmission cycles
Source: Sci Rep. 2018 Mar 7;8:4140. doi: 10.1038/s41598-018-22455-x (PMC5841364; doi:10.1038/s41598-018-22455-x)
Supplement: Supplementary file 1 — Supplementary materials [file 41598_2018_22455_MOESM1_ESM.pdf]

Detailed ecological associations of triatomines revealed by metabarcoding and next-generation sequencing: implications for triatomine behavior and *Trypanosoma cruzi* transmission cycles

Eric Dumonteil[1,2,*], Maria-Jesus Ramirez-Sierra[2], Silvia Pérez-Carrillo[2], Christian Teh-Poot[2], Claudia Herrera[1], Sébastien Gourbière[3] & Etienne Waleckx[2]

Supplementary materials:

Supplementary Figure S1. Rarefaction curve of *T. dimidiata* gut microbiome order diversity.

Supplementary Figure S2: Neighborhood connectivity distribution of *T. dimidiata* feeding network.

Supplementary Table 1: Biological information and number of sequence reads obtained from *Triatoma dimidiata* specimens

Supplementary Table 2: Species identification by MEGABLAST of 12S rRNA sequences.

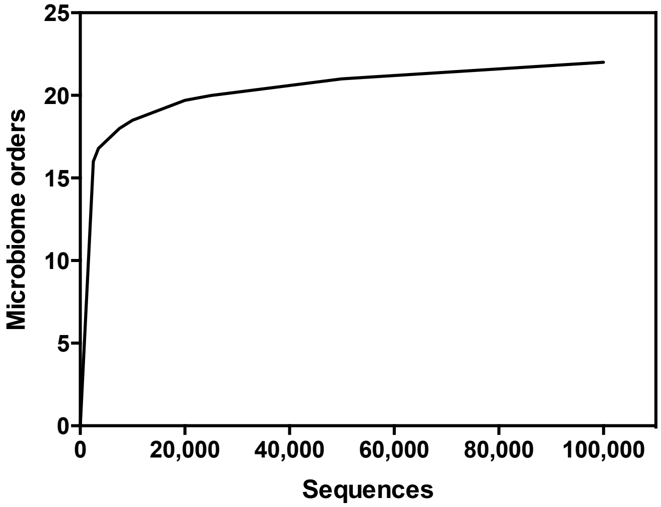

Supplementary Figure S1. Rarefaction curve of *T. dimidiata* gut microbiome order diversity.
Bacterial orders were plotted against the number of partial 16s rARN sequences analyzed to assess sequence coverage of the microbiome diversity. The observed plateau suggested that the majority of the diversity had been accounted for with our sampling.

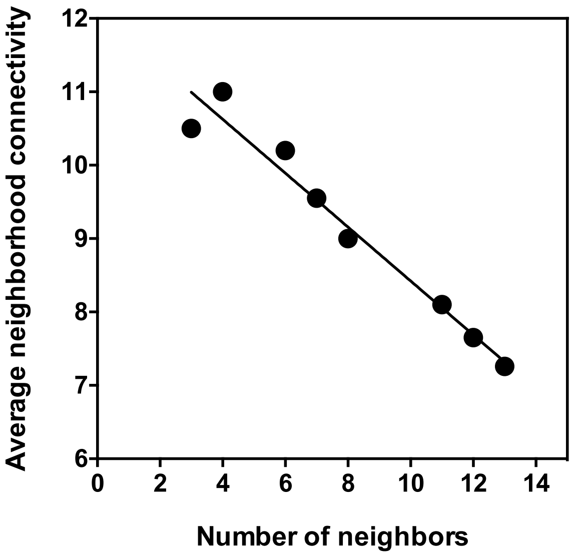

Supplementary Figure S2: Neighborhood connectivity distribution of *T. dimidiata* feeding network.
The network shown in Fig. 4 was analyzed in Cytoscape. The average connectivity of all neighbors of a node represents the neighborhood connectivity. The neighborhood connectivity distribution shows the average of the neighborhood connectivity of all nodes with their respective neighbors. Neighborhood connectivity decreased strongly with the number of neighbors (R^2 = 0.96, P < 0.0001), indicating that highly connected nodes predominate in shaping the network.

Supplementary Table 1: Biological information and number of sequence reads obtained from *Triatoma dimidiata* specimens

**Bug ID**	Bok006	Bok011	Bok012	Sud036	Sud054	Sud059	Sud061	Sud094	Sud099	Tey011	Tey012	Tey015	Tey016	Tey139	TOTAL
**Locality**	Bokoba	Bokoba	Bokoba	Sudzal	Sudzal	Sudzal	Sudzal	Sudzal	Sudzal	Teya	Teya	Teya	Teya	Teya	-
**Sex**	Female	Female	Female	Male	Female	Male	Male	Male	Male	Male	Female	Female	N5	Male	-
**Habitat**	Sylvatic	Dom.	Dom.	Dom.	Sylvatic	Sylvatic	Sylvatic	Sylvatic	Sylvatic	Dom.	Dom.	Dom.	Dom.	Dom.	-
***Triatoma dimidiata* ITS2**	11,491	31,748	11,750	15,320	17,994	15,619	41,014	11,800	25,226	23,930	10,960	14,964	13,474	15,493	**260,783**
**Vertebrate 12S**	15,328	50,312	16,870	51,849	1,005	1,157	1,025	3,689	62,181	4,165	1,145	1,130	2,115	6,059	**218,030**
**Microbial 16S**	25,748	18,985	2,390	18,024	4,720	8,576	10,859	10,518	339,161	35,730	7,068	3,719	-	1,800	**487,298**
***Trypanosoma cruzi* mini exon**	-	-	3,604	9,659	-	-	-	-	-	130	-	13,947	9,407	13,775	**50,522**

Dom: domestic

Supplementary Table 2: Species identification by MEGABLAST of 12S rRNA sequences.

Bug ID	Sequence length	% identity	E value	Frequency (%)	Species match	Likely host species reported in the Yucatan
Bok006	177	100	6.95E-84	55.4	*Bos taurus*	*Bos taurus*
Bok006	176	100	2.41E-83	0.6	*Canis lupus*	*Canis lupus familiaris*
Bok006	173	100	1.00E-81	27.2	*Homo sapiens*	*Homo sapiens*
Bok006	180	100	1.67E-85	<0.5	*Mus musculus*	*Mus musculus*
Bok006	179	99.4	2.98E-82	0.5	*Gallus gallus*	*Gallus gallus*
Bok006	177	99.4	2.95E-82	0.7	*Meleagris gallopavo*	*Meleagris gallopavo*
Bok006	176	99.4	1.02E-81	<0.5	*Sciurus variegatoides*	*Sciurus yucatanensis/sciurus deppei*
Bok006	179	96.6	8.04E-77	15.4	*Zenaida macrura/Columba livia*	*Zenaida* spp.*/Columba livia*
Bok011	177	100	6.98E-84	0.9	*Canis lupus*	*Canis lupus familiaris*
Bok011	173	100	1.00E-81	99.1	*Homo sapiens*	*Homo sapiens*
Bok012	177	100	6.98E-84	95.3	*Canis lupus*	*Canis lupus familiaris*
Bok012	177	100	2.41E-83	1.1	*Canis lupus*	*Canis lupus familiaris*
Bok012	178	100	2.02E-84	0.6	*Felis catus/felis silvestris*	*Felis catus*
Bok012	173	100	1.00E-81	0.5	*Homo sapiens*	*Homo sapiens*
Bok012	177	99.4	3.57E-81	1.0	*Canis lupus*	*Canis lupus familiaris*
Bok012	176	99.4	1.02E-81	0.8	*Canis lupus*	*Canis lupus familiaris*
Bok012	177	99.4	2.95E-82	<0.5	*Rattus rattus*	*Rattus rattus*
Sud036	173	100	3.47E-81	100.0	*Homo sapiens*	*Homo sapiens*
Sud054	177	100	6.95E-84	<0.5	*Bos taurus*	*Bos taurus*
Sud054	173	100	1.00E-81	92.8	*Homo sapiens*	*Homo sapiens*
Sud054	176	100	2.41E-83	2.0	*Sus scrofa*	*Sus scrofa*
Sud054	174	99.4	1.23E-80	3.4	*Coendou bicolor*	*Coendou mexicanus*
Sud054	173	96.5	1.39E-73	1.7	*Homo sapiens*	*Homo sapiens*
Sud059	176	100	2.41E-83	1.4	*Canis lupus*	*Canis lupus familiaris*

Sud059	173	100	1.00E-81	78.4	*Homo sapiens*	*Homo sapiens*
Sud059	178	100	1.67E-85	2.2	*Mus musculus*	*Mus musculus*
Sud059	168	97.4	6.09E-72	0.9	*Potos flavus*	*Potos flavus*
Sud059	179	96.6	8.04E-77	3.0	*Zenaida macrura /Columba livia*	*Zenaida* spp./*Columba livia*
Sud061	173	100	1.00E-81	84.4	*Homo sapiens*	*Homo sapiens*
Sud061	172	100	3.47E-81	4.0	*Homo sapiens*	*Homo sapiens*
Sud094	176	100	2.41E-83	7.0	*Canis lupus*	*Canis lupus familiaris*
Sud094	172	100	3.47E-81	36.6	*Homo sapiens*	*Homo sapiens*
Sud094	173	100	1.00E-81	0.8	*Homo sapiens*	*Homo sapiens*
Sud094	178	100	2.00E-84	0.9	*Mus musculus*	*Mus musculus*
Sud094	174	99.4	1.23E-80	54.1	*Coendou bicolor*	*Coendou mexicanus*
Sud094	174	98.9	1.81E-78	0.5	*Coendou bicolor/Coendou prehensilis*	*Coendou mexicanus*
Sud099	173	100	3.47E-81	0.5	*Homo sapiens*	*Homo sapiens*
Sud099	176	99.4	1.02E-81	97.7	*Sciurus variegatoides*	*Sciurus yucatanensis/Sciurus deppei*
Sud099	172	98.9	1.02E-81	1.9	*Syntheosciurus brochus*	*Sciurus yucatanensis/Sciurus deppei*
Tey011	173	100	2.46E-83	57.8	*Homo sapiens*	*Homo sapiens*
Tey011	177	100	6.95E-84	35.5	*Bos taurus*	*Bos taurus*
Tey011	176	100	2.41E-83	1.0	*Canis lupus*	*Canis lupus familiaris*
Tey011	173	100	2.46E-83	0.5	*Homo sapiens*	*Homo sapiens*
Tey011	178	100	2.00E-84	7.0	*Mus musculus*	*Mus musculus*
Tey011	177	100	6.95E-84	0.5	*Rattus norvegicus*	*Rattus* spp.
Tey011	174	99.4	1.23E-80	1.4	*Coendou bicolor*	*Coendou mexicanus*
Tey011	177	97.7	1.86E-78	0.7	*Artibeus lituratus/Artibeus jamaicensis*	*Artibeus lituratus/Artibeus jamaicensis*
Tey012	176	100	2.41E-83	30.7	*Canis lupus*	*Canis lupus familiaris*
Tey012	173	100	1.00E-81	64.8	*Homo sapiens*	*Homo sapiens*
Tey012	178	100	2.00E-84	2.3	*Mus musculus*	*Mus musculus*

Tey012	177	97.7	1.86E-78	0.9	*Artibeus lituratus/Artibeus jamaicensis*	*Artibeus lituratus/Artibeus jamaicensis*
Tey015	176	100	2.41E-83	1.6	*Bos taurus*	*Bos taurus*
Tey015	177	97.2	7.93E-77	0.8	*Artibeus lituratus/Artibeus jamaicensis*	*Artibeus lituratus/Artibeus jamaicensis*
Tey015	173	100	1.00E-81	85.4	*Homo sapiens*	*Homo sapiens*
Tey016	173	100	5.88E-84	25.0	*Homo sapiens*	*Homo sapiens*
Tey016	179	99.4	2.41E-83	50.0	*Canis lupus*	*Canis lupus familiaris*
Tey016	149	97.7	1.02E-81	25.0	*Sciurus variegatoides*	*Sciurus yucatanensis/Sciurus deppei*
Tey139	176	100	2.41E-83	3.9	*Canis lupus*	*Canis lupus familiaris*
Tey139	173	100	1.00E-81	92.4	*Homo sapiens*	*Homo sapiens*
Tey139	173	100	1.00E-81	3.7	*Homo sapiens*	*Homo sapiens*

In some cases, multiple sequences from the same bug corresponded to the same host species, but with a different haplotypes.
